# The Better-Than-Average Effect Is Observed Because “Average” Is Often Construed as Below-Median Ability

**DOI:** 10.3389/fpsyg.2017.00898

**Published:** 2017-06-22

**Authors:** Young-Hoon Kim, Heewon Kwon, Chi-Yue Chiu

**Affiliations:** ^1^Department of Psychology, Yonsei UniversitySeoul, South Korea; ^2^Department of Psychology, The Chinese University of Hong KongHong Kong, Hong Kong

**Keywords:** better-than-average effect, average, information theory, self-enhancement, social comparison

## Abstract

Most people rate their abilities as better than “average” even though it is statistically impossible for most people to have better-than-median abilities. Some investigators explained this phenomenon in terms of a self-enhancement bias. The present study complements this motivational explanation with the parsimonious cognitive explanation that the phrase “average ability” may be interpreted as below-median ability rather than median ability. We believe people tend to construe an “average” target that is based on the most representative exemplar, and this result in different levels of “average” in different domains. Participants compared their abilities to those of an average person, typical person, and a person whose abilities are at the 40th, 50th, or 60th percentile. We found that participants’ interpretation of “average” ability depended on the perceived difficulty of the ability. For abilities perceived as easy (e.g., spoken and written expression), participants construed an “average” target at the 40th percentile (i.e., below-median ability) and showed a marked better-than-average effect. On the contrary, for abilities perceived to be difficult, participants construed an “average” target at the median or even above the median.

## Introduction

Research in social judgment has promoted the view that most people are unrealistic self-enhancers. A robust, frequently cited research finding in support of this view is the better-than-average effect (BAE). At least in Western cultures, across age groups, occupations, and ability domains, when asked to evaluate their abilities, most people say they are *better than average* (College Board, 1976–1977; Cross, 1977; Alicke et al., 1995). As it is statistically impossible that more than 50 percent of a population are better than average in any ability when the average person’s ability is at the 50th percentile, the robust BAE seems to suggest that most people possess positive but unrealistic self-perceptions (Taylor and Brown, 1988).

The present study takes a different perspective on the BAE. Specifically, we contend that although it is impossible for most people to possess above median ability in any ability domain, it is possible for most people to have better-than-average ability, if the term “average” is not interpreted as “median,” as most of the previous literature has assumed. In this scenario, people may see the comparison target (*the average person*) not as the statistical mean or median but as someone with below-median ability or in other words, mediocre ability. We believe that when trying to conjure up an average, people choose a target they believe is the most representative of the group, and this comparison target is more often than not someone with below-median ability (Maguire et al., 2016), particularly in the traditionally measured ability domains in the BAE literature. In other words, the BAE may not be an accurate reflection of self-enhancement bias, if people perceive “average” not as a neutral statistical term but as a slightly negative term connoting mediocrity, found somewhere below median. To flesh out this idea, in the following sections we review the major theoretical accounts of the BAE and the hypotheses of this study.

## Major Theoretical Accounts

Building on the premise that it is statistically impossible for most people to have better-than-average abilities when the average person’s ability is at the 50th percentile, researchers have interpreted the BAE as a bias in social comparative judgments. One view is that the BAE results from people’s cognitive biases, such as egocentrism and focalism (for a complete review, see Chambers and Windschitl, 2004). According to this explanation, when people are asked to make comparative judgments of ability (e.g., how intelligent are you relative to an average student in your school?), they are less likely to use information about the reference target (the average student) than information about the self (Weinstein, 1980; Weinstein and Lachendro, 1982; Kruger, 1999; Kruger and Dunning, 1999; Chambers et al., 2003), because information about the self is more salient and more likely to receive focal attention (Windschitl et al., 2003; Chambers and Suls, 2007). Consistent with this argument, research has shown that people tend to judge their own ability favorably relative to others when they find the ability task to be easy (without considering that others will find it easy too) and unfavorably when they find the task to be difficult (without considering that others will find it difficult too; College Board, 1976–1977; Kruger, 1999).

Another widely accepted account of the BAE claims that people are motivated to self-enhance because a positive (albeit unrealistic) view of the self gives rise to positive feelings and serves important self-protective functions (Sedikides and Strube, 1997). In the view of many researchers, the BAE reflects the motivation to see oneself in a positive way above and beyond the abovementioned cognitive biases, because BAE is higher for important attributes than for unimportant ones and increases after a threat to one’s self-worth is experienced (Brown, 2012). The BAE has been found to be related to better psychological health, including higher self-esteem, lower depression (Taylor and Brown, 1988; Brown and Dutton, 1995), and better intellectual functioning (Swann et al., 1989).

In short, both the cognitive and motivational accounts assume that the BAE represents a judgment bias at the group level. We agree that both cognitive biases and self-enhancement motivation can contribute to the tendency to evaluate one’s relative abilities favorably, particularly for basic abilities or skills (e.g., driving). However, we contend that the BAE may not solely be attributable to self-enhancement, although this is widely accepted.

Specifically, we argue that when comparing one’s abilities or skills with those of an average person, people may not use someone with mean or median ability as the comparison target. The word “average” has multiple meanings: It may refer to (1) a statistical average such as the arithmetic mean or median (e.g., “The average height of a 10-year-old girl in 1963 was about 55.5 inches”), (2) an ordinary, typical standard (e.g., “The average American prefers driving to taking public transportation”), and (3) a mediocre, or relatively low standard (e.g., “He is a very average director”).

Computing the representation of a statistical average exemplar is a cognitively demanding task—it requires attention to the relevant sampling space, which may often be obscure when judging covert abilities, and consideration of possible sampling biases in relation to sample size, population homogeneity, sampling methods, and so on (Nisbett et al., 1983). Hence, it is unlikely that people have pre-constructed, pre-stored, and accurate statistical average exemplars for various abilities in their memory. It is also unlikely that people would be able to spontaneously compute accurate statistical average exemplars online and use them successfully when asked to make quick relative judgments of ability. Many researchers have struggled with the term “average.” Although the statistical mean or median might seem to be the most representative average, the statistical mean or median no longer stands as a representative standard when there is only limited information and/or the data is skewed. According to information theory, “representativeness” converges to the exemplar that holds the most meaningful information (Maguire et al., 2016). For example, researchers who construct house price indices can sometimes find that their “average” house price is, in fact, less than both the statistical mean price and the median house price. This is because their techniques focus on the “typical” house, the one whose price conveys the most information about all other houses. We believe that just as researchers try to make an inference of the most representative average based on partial information, laypeople do the same when making social comparisons. In other words, given the difficulty of computing the exemplar of the statistical average and the effort to conjure the most logical average carrying the most information, the average that contains the most information would differ depending on the domain, and, more often than not, would differ from the statistical mean or median.

Thus, we argue that when rendering comparative judgments of ability, people are likely to employ typical (representative) exemplars that are most cognitively available at the moment of the judgment (Tversky and Kahneman, 1974; Nisbett et al., 1983) and hold the most information (Maguire et al., 2016). One might believe that people would take the most frequently occurring exemplar as the representative sample but this is not the case. As the computation of mean or median is difficult, so is the statistical mode, and the perceived representative exemplar does not necessarily mean the most frequent exemplar. Although it is difficult to predict exactly who is believed to be the most representative exemplar by people, past literature has suggested this to be someone with rather lower ability in easy tasks (Harris and Middleton, 1994; Kruger, 1999). Moreover, we believe that this typical exemplar tends to vary with the nature of the ability domain. Specifically, when the ability domain requires elementary skills only (e.g., driving, getting along with others, sales, and verbal skills, i.e., those ability domains whose tasks are relatively easy to perform), the cognitively available, typical exemplar would be a person with below-median ability. For example, because it does not require intensive training to become a driver and most people drive, when a college student compares her/his driving ability with the average student, the image of the comparison target that readily comes to mind is likely to be one of a typical (representative) student with below-median driving ability. This is because the actual median point is much higher than participants’ perception. This tendency to employ a typical exemplar with below-median ability will disappear when people make comparative judgments in a domain of ability that is relatively difficult to perform (e.g., acting, music, art, mechanics, or science), requires intensive training and/or is limited to a more select population.

Prior research supports this prediction, particularly when people are directly asked to make comparative judgments. That is, when people are asked to compare themselves with “the average person” in ability domains that are relatively easy to perform, they interpret “the average person” pejoratively, as possessing mediocre or low ability or performance (Perloff and Fetzer, 1986; Harris and Middleton, 1994). For example, Perloff and Fetzer (1986) found that when participants were asked to compare themselves with “the average person,” they selected a target who performed relatively unfavorably on the dimension assessed. These all support our hypothesis that people conjure an average person with below-median ability in easy ability domains, and an average person closer to the median or even better than median in difficult ability domains.

## Objectives of the Present Study

In the present study, we addressed four related issues. To test the first hypothesis, that participants use a typical exemplar to mentally simulate an average exemplar, we had one group of students compare their abilities to those of *an average student* on campus, and another group of students compare their abilities to *a typical student* on campus. We expected ratings in the average target condition not to differ from those in the typical target condition.

*Hypothesis 1*: *The ratings of the average student condition will not be different from those of the typical student condition* (H1).

Second, to determine the level of relative ability participants attribute to the average student, we had another three groups of participants compare their own abilities to a student whose abilities were at the 40th, 50th, or 60th percentile. Ratings from these conditions would allow us to infer who the average student was in the participants’ mind when they performed the comparative ability judgments. For example, if it turned out that comparative judgments in the average student condition did not differ from those in the typical student condition and the 40th percentile student condition but were better than those in the 50th and 60th percentile, this would suggest that in the mind of the participants, an average student was a typical student with below-median ability.

Third, by observing how participants rate themselves relative to the 50th percentile student, we could infer whether the participants as a group inflated their relative ability ratings. If it turned out that participants rated their abilities as higher than those of the 50th percentile student (hereafter referred to as the better-than-median effect), we would be able to conclude with confidence the presence of a self-enhancement bias at the group level.

Fourth, to test the hypothesis that the tendency to view a typical exemplar as someone with below-median ability would be particularly pronounced when participants make comparative judgments in easy ability domains, we had each participant rate all the 14 abilities covered in the College Board (1976–1977) survey that have sizable variation in perceived easiness. As discussed earlier, there is a robust relationship between the perceived difficulty of a domain and the BAE. For example, for the 14 ability domains used in the College Board survey, Kruger (1999) found a highly significant relationship between domain difficulty and the percentage of participants rating themselves as better than average in that domain, *r* = -0.81, *p* < 0.001. That is, the BAE was particularly pronounced for general ability domains perceived to be easy by participants, such as getting along with others, spoken expression, written expression, creative writing, and leadership. In contrast, the BAE was not observed for artistic ability domains that were perceived to be most difficult, such as art, acting, and music. For scientific ability domains that were perceived as moderately difficult, such as mechanics and science, the BAE was found to be weak. We reasoned that this pattern was observed partly because in easy ability domains people construe the typical, average person as lower in ability than in difficult ability domains.

With the three latter hypotheses, we expected different patterns for the three different ability domains as follows:

*Hypothesis 2a*: *For general abilities, which would be perceived as easy abilities, people’s perception of an “average” target will be a target at the 40th percentile* (H2a).
*Hypothesis 2b*: *For scientific abilities, which would be perceived as abilities with medium difficulty, people’s perception of an “average” target will be a target at the 50th percentile* (H2b).
*Hypothesis 2c*: *For artistic abilities, which would be perceived as difficult abilities, people’s perception of an “average” target will be a target at the 60th percentile* (H2c).

## Materials and Methods

### Ethics Statement

The study was approved by the Institutional Review Board for the Protection of Human Subjects at the University of Illinois at Urbana–Champaign. All participants voluntarily filled out an informed consent form agreeing to participate in the study.

### Participants

In all, 288 participants (144 males) were recruited from a public university in the United States. The mean age of the participants was 18.91 years old with a standard deviation of 0.96. Participants received extra credit toward their class for their participation.

### Materials and Procedures

We had participants evaluate themselves on 14 abilities and skills (ability to get along with others, spoken expression, written expression, creative writing, leadership, sale, organizing for work, athletics, science, mathematics, mechanics, acting, music, and art) covered in the College Board (1976–1977) survey. The abilities were rated in the order that the College Board survey was conducted, from the ability to get along with others to mechanics (College Board, 1976–1977). There were five between-subjects conditions in the experiment. In the average target condition, participants rated their own abilities and skills relative to those of an average student on campus on a 7-point Likert scale ranging from 1 (*much worse than*) to 4 (*as good as*) and 7 (*much better than*). A mean rating greater than 4 in a particular ability or skill would be taken to indicate the presence of the BAE (as conventionally defined in the literature) on this ability/skill. A total of 84 participants (43 males) were in this condition.

To determine whether the *average student* was perceived the same as a typical student, we included a typical student condition, in which participants rated their abilities/skills relative to those of a typical student on campus. To further determine what percentile *average ability or skill* refers to, we had three groups of participants rate themselves on each ability or skill relative to a target person whose ability or skill fell exactly on the 40th percentile, 50th percentile, or 60th percentile. Specifically, for each ability or skill, participants in the 50th (40th or 60th) percentile condition were asked to think of a person whose ability or skill was better than 50% (40 or 60%) of the students and worse than 50% (60 or 40%) of the students on campus. Next, the participants were instructed to think about who this person could be among the students they know, and rate their own ability or skill relative to this target. To the typical student condition, 94 participants (41 males) were assigned, while others were assigned to the 40% (32 participants, 20 males), 50% (39 participants, 21 males), and 60% (39 participants, 19 males) condition. Since the focus of the present study was to examine how differently or similarly people perceived an average student and a typical student, we collected more than 80 participants for each condition to fully detect the difference based on the expected effect size. Moreover, to examine where the average or typical student stands in terms of statistical percentiles, the 40th, 50th, and 60th percentile condition obtained the minimal number of participants that could be compared with the other conditions. Participants in all five experimental conditions indicated their responses on the same 7-point Likert scale.

## Results

### Was There a Significant BAE?

The only gender difference we found in our analyses was men’s tendency to rate themselves more favorably in scientific abilities, *F*(1,82) = 21.81, *p* < 0.001, $\eta_{p}^{2}$ = 0.18. Thus, we did not include gender in the analyses reported below. Consistent with past findings, significant BAE was observed in most abilities and skills. In the average student condition, in which participants compared themselves to an average student, the mean ratings of the 14 abilities and skills ranged from 5.27 (ability to get along with others) to 3.60 (mechanics). The mean ratings for nine abilities and skills (ability to get along with others, written expression, spoken expression, leadership, mathematics, creative writing, science, organizing for work, and athletics) were significantly greater than 4.0 (the mid-point of the scale), *ps* < 0.05. A significant worse-than-average effect was found for acting and mechanics, *ps* < 0.05.

**FIGURE 1:**
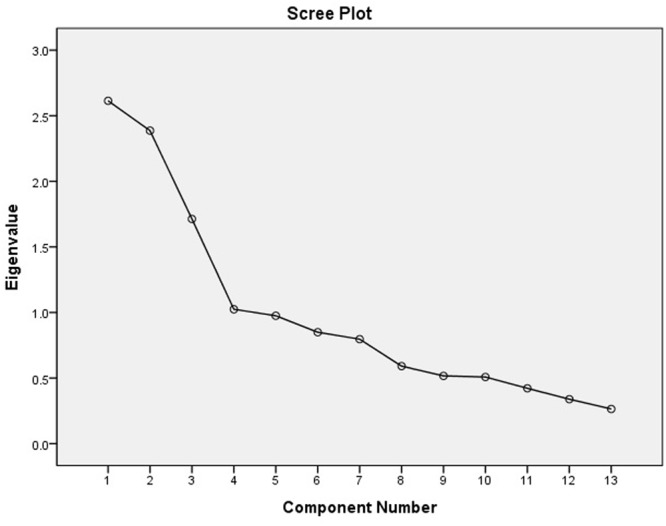
Scree plot supporting a three-factor solution (data obtained from the present study).

To simplify subsequent analyses, we pooled the data from all experimental conditions and performed a principal component analysis on the 14 ability and skill ratings, a statistical method that reduces the number of variables to a smaller number of components, to simplify subsequent analyses. One of the widely used criteria for determining the number of components (Velicer and Jackson, 1990) is extracting principal components with eigenvalues greater than one, also known as principal component analysis. Another useful criterion is running a scree test that plots the components on the *x*-axis and the corresponding eigenvalues on the *y*-axis in descending order of their eigenvalues and retaining the components that fall on the steep curve before the first point that starts the flat line trend, called the elbow. We show a scree plot demonstration in **Figure 1** on the basis of our data, which contains an “elbow” after the third factor, supporting a three-factor solution (Reise et al., 2000). Based on these two criteria, we were able to retain three principal components that had eigenvalues greater than one and fell on the steep curve. In Kruger’s (1999) research, he distinguished the difficulty level of various abilities where general, scientific, and artistic abilities were rated easy, moderate, and difficult, respectively. As our three orthogonal principal components could be described well as general, scientific, and artistic (see the component loadings in **Table 1**), we inferred that they represent abilities perceived as easy, moderate, and difficult, respectively. The first component accounted for 19.4% of the total variance and had significant post-orthogonal rotation (the Verimax with Kaiser Normalization) loadings (>0.40) from the linguistic (spoken expression, written expression, creative writing), interpersonal (ability to get along with others, leadership, sale), self-management (organizing for work), and kinesthetic (athletics) ability domains. We used the unweighted means of these eight items to form a general ability component (α = 0.69). These ability domains were perceived as relatively easy (Kruger, 1999). The second component had significant loadings (>0.60) from the three science-related abilities (science, mathematics, and mechanics) and accounted for 17.0% of the total variance. We formed the scientific ability component by taking the unweighted average of the three items (α = 0.70). These ability domains were perceived as moderately difficult (Kruger, 1999). The three art-related abilities (acting, music, art) had significant loadings (>0.70) on the third component and accounted for 15.2% of the total variance. The unweighted average of these three items was used to create the artistic ability component (α = 0.66). These ability domains were perceived as very difficult (Kruger, 1999). The three principal components together accounted for 51.6% of the total variance (see **Tables 1, 2**). The amount of variance explained by the three principal components is relatively low, which is a limitation of the present study.

**Table 1:** Factor loadings based on principal components analysis with varimax rotation for 14 items.

	General	Scientific	Artistic
Written expression	**0.653**	–0.418	0.115
Spoken expression	**0.707**	0.003	0.101
Leadership	**0.775**	0.146	–0.013
Creative writing	**0.549**	–0.498	0.271
Organizing for work	**0.406**	0.078	–0.216
Athletics	**0.463**	0.168	–0.178
Sale	**0.453**	0.268	0.170
Science	0.097	**0.758**	–0.119
Math	0.096	**0.764**	–0.123
Mechanics	0.111	**0.640**	0.204
Music	–0.089	0.179	**0.765**
Art	–0.128	–0.249	**0.739**
Acting	0.232	–0.047	**0.756**

*^∗^Values in bold type indicate the primary factor on which a given item loaded.*

**Table 2:** Eigenvalues rotation sums of squared loadings.

Component	Total	% of variance	Cumulative %
1 (General)	2.521	19.390	19.390
2 (Scientific)	2.212	17.019	36.409
3 (Artistic)	1.979	15.227	51.636

Next, we tested whether there was a significant BAE in the three ability domains. In the average student condition, the mean ratings were greater than 4 for general abilities (*M* = 4.78, *SD* = 0.64; *t*(83) = 11.25, *p* < 0.001, *d* = 1.23, 95% confidence interval (CI) = [0.94, 1.51]) and scientific abilities (*M* = 4.43, *SD* = 1.04; *t*(83) = 3.85, *p* < 0.001, *d* = 0.42, 95% CI = [0.20, 0.64]). The mean rating for artistic abilities (*M* = 3.81, *SD* = 1.13) was not significantly different from 4, *t*(83) = -1.52, *p* = 0.13.

### Who Was the Average Student?

We performed a multivariate analysis of variance on the ratings in the three ability domains to see whether the main effect of condition (five experimental conditions) is reliably different across the three ability domains. The main effect of condition was significant, multivariate *F*(12,834) = 3.68, *p* < 0.001, $\eta_{p}^{2}$ = 0.05. To clarify this multivariate effect, we performed a one-factor ANOVA on each of the three ability domains with condition as the between-subjects factor. We used the average student condition as the reference condition in a set of simultaneous simple contrasts. Moreover, to adjust the alpha level, each of the independent sample *t*-tests were compared with an adjusted α level of 0.0125 (the conventional 0.05 divided by 4, since there are four comparisons with the average student condition) and the one-sample *t*-tests were compared with an adjusted α level of 0.01 (the conventional 0.05 divided by 5 because there are five comparisons with 4.0).

**FIGURE 2:**
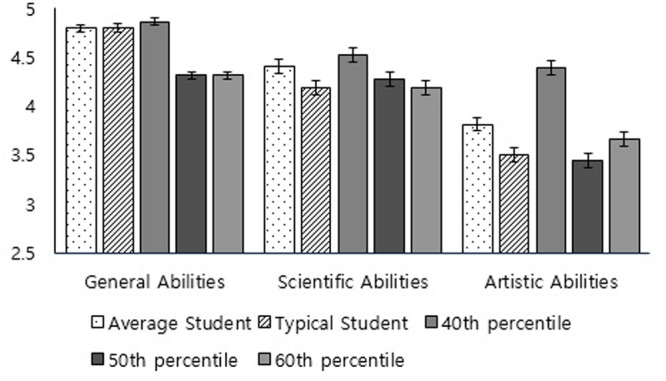
Mean rating of one’s ability relative to different comparison targets in 3 ability domains.

For general abilities, the main effect of condition was significant, *F*(4,283) = 6.83, *p* < 0.001, $\eta_{p}^{2}$ = 0.09. As illustrated in **Figure 2**, participants’ mean rating in the average student condition (*M* = 4.79, *SD* = 0.64) was not significantly different from the typical student condition (*M* = 4.80, *SD* = 0.69), *t*(172) = 0.26, *p* = 0.87, *d* = -0.01, 95% CI = [-0.31, 0.28] confirming our first hypothesis (H1) that the average student would be regarded as a typical student, or the 40th percentile target condition (*M* = 4.86, *SD* = 0.85), *t*(113) = 0.51, *p* = 0.66, *d* = -0.10, 95% CI = [-0.51, 0.31] but was significantly higher than that in the 50th percentile target condition (*M* = 4.31, *SD* = 0.85), *t*(119) = -3.40, *p* < 0.001, *d* = 0.67, 95% CI = [0.28, 1.06] and the 60th percentile target condition (*M* = 4.31, *SD* = 0.70), *t*(120) = -3.38, *p* < 0.001, *d* = 0.72, 95% CI = [0.33, 1.11]. Thus, although we obtained a significant traditionally defined BAE in general abilities, in the participants’ mind, the average student was less able than the statistical average (50th percentile) and was instead similar to a typical student with below-median abilities (40th percentile), confirming our second hypothesis (H2a).

For scientific abilities, the main effect of condition was not significant, *F*(4,283) = 0.82, *p* = 0.52, $\eta_{p}^{2}$ = 0.01. The mean rating in the average student condition did not differ from that in the other four conditions, which only partially confirmed our hypotheses. More specifically, it confirmed that the average student was not different from the typical student, confirming H1, but this average student did not specifically match the 50th percentile target. Planned analysis revealed a significant traditionally defined BAE in this ability domain (*M* = 4.43, *SD* = 1.04), *t*(83) = 3.85, *p* < 0.001, *d* = 0.42, 95% CI = [0.20, 0.64]. However, the participants did not rate their scientific abilities as better than those of the typical student (*M* = 4.18, *SD* = 1.28), *t*(93) = 1.40, *p* = 0.17, *d* = 0.14, 95% CI = [-0.06, 0.35], a 40th percentile target (*M* = 4.52, *SD* = 1.26), *t*(32) = 2.35, *p* = 0.026, *d* = 0.41, 95% CI = [-0.06, 0.87], a 50th percentile target (*M* = 4.27, *SD* = 1.38), *t*(38) = 1.20 *p* = 0.24, *d* = 0.19, 95% CI = [-0.13, 0.51], or a 60th percentile target (*M* = 4.18, *SD* = 1.35), *t*(38) = 0.83, *p* = 0.41, *d* = 0.13, 95% CI = [-0.18, 0.44]. In spite of our hypothesis regarding the scientific ability domain (H2b), it was rather difficult to conclude that participants regarded the average student as someone at the 50th percentile target.

For artistic abilities, the main effect of condition was significant, *F*(4,283) = 3.58, *p* = 0.007, $\eta_{p}^{2}$ = 0.05. However, despite the planned analyses showed significant difference for the conventional α level (0.05), they did not reach significant difference for the adjusted α level (0.0125). Therefore, there was no difference between the mean rating in the average student condition (*M* = 3.81, *SD* = 1.13) and that in the typical student condition (*M* = 3.50, *SD* = 1.26), *t*(176) = -1.65, *p* = 0.10, *d* = 0.26, 95% CI = [-0.04, 0.55], the 40th percentile target condition (*M* = 4.39, *SD* = 1.46), *t*(114) = 2.18, *p* = 0.03, *d* = -0.47, 95% CI = [-1.01, 0.07], the 50th percentile target condition (*M* = 3.44, *SD* = 1.30), *t*(121) = -1.54, *p* = 0.12, *d* = 0.31, 95% CI = [-0.07, 0.69], as well as the 60th percentile target condition (*M* = 3.66, *SD* = 1.34), *t*(121) = -0.81, *p* = 0.42, *d* = 0.12, 95% CI = [-0.26, 0.50]. In short, for artistic abilities, participants did not display the traditionally defined BAE. Moreover, in the participants’ mind, a student with average artistic abilities was a typical student as we expected in our first hypothesis (H1), but unlike our second hypothesis regarding the artistic ability domain (H2c), it was difficult to conclude this student is someone with above median artistic abilities.

## Discussion

Past studies have focused mainly on why people put themselves above average and how the information of the self contributes to the BAE. However, not much attention was paid to how people interpret the term “average” and how the information is processed regarding it. More specifically, past literature has assumed that the term “average” is the statistical average: the mean. Nonetheless, our study explored who people really considered to be “the average person” in social comparisons and showed how people conjure up an average person different from the statistical average or median, thereby contributing to the strengthening of the BAE.

Replicating past findings, we observed the BAE in the evaluation of both general and scientific abilities. However, the participants rated their abilities as better than median in the domain of general abilities only. More importantly, our results indicate that the BAE might not be an accurate indicator of self-enhancement bias in social comparisons. Although we obtained a significant BAE in general abilities, participants did not see the average student as having median abilities in this domain. Instead, participants viewed the average student as a typical student with below-median abilities. We obtained the same results in the domain of scientific abilities. This result indicates that when people say that they are better than average in general or scientific abilities, what they wish to communicate is that their abilities are better than someone with below-median abilities, who they believe is the most representative comparison target in those domains. This leads the authors to infer that when people say that they are better than average, they might be correct. People, in fact, want to be correct, and the effort to conjure the most representative “average” made them seem to self-enhance more than they actually do. The decrease in BAE according to the increase in difficulty of the ability domain further supports the authors’ claim that people employ someone as an average comparison target who is most representative.

Thus, when assessing self-enhancement bias in comparative judgments of ability, it is important to ascertain how the judges interpret “average ability” and accordingly interpret the results with caution. When asked to compare their ability to an average person, some people may not grasp the intended meaning of the comparison target (e.g., median ability). Indeed, as studies have shown, when people are asked to compare their abilities to those of a vivid and specific, rather than general, comparison target, the BAE diminishes (Weinstein, 1980; Klar and Giladi, 1997). Consistent with this idea, in the current study, the better-than-median effect was much smaller than the BAE. In addition, consistent with past research, the present study found that if the ability under discussion is perceived to be in the easy domain, the participants showed a stronger BAE; conversely, if the ability was difficult, the participants showed a weaker BAE.

The present study is not without its limitations. While the average student and the typical student conditions asked participants to make social comparisons with a rather abstract person who has average ability, participants in the three percentile conditions were asked to think of a specific person they knew, which was presumably a more concrete comparison target. This was done to make it easy for the participants to conjure the comparison target, because this is not something people do in daily situations, whereas comparing oneself to an average person is a more familiar task. However, this might have unintentionally caused different levels of abstractness of the target and thus worked as a confounding factor. More specifically, concrete targets are known to decrease the BAE because people tend to rate concrete comparison targets more favorably than abstract ones (Alicke et al., 1995). Therefore, the study’s results might have been affected by this different level of abstractness. Furthermore, all participants were college students, which is a sample with better-than-median talent and education, and there may be a stronger BAE in general among such a population. However, participants were asked to conjure someone within their college, and thus the comparison target was not based on the general population. Moreover, if participants have a tendency to view themselves as better than average, this rather strengthens our point that even people who are in general better than median in their talent and education, are not conjuring someone at the 50th percentile but rather someone lower than the median, at least in easy ability domains. Nonetheless, because of the possible confounding effect of the different level of abstractness and the unique characteristics of the college student sample, replication in future studies with a uniform level of abstractness of the comparison target and with a more general population would strengthen the results of this study. Moreover, although the present study calls for caution in the interpretation of the BAE, the reasons for this cognitive phenomenon, people perceiving “average” to be below-median, is not covered by the scope of the present study. It may be that the reasons for people’s perception are to be traced to a motivational explanation. For easy tasks, it might be embarrassing to admit that one is below-median, whereas for difficult tasks, such embarrassment would not necessarily be present. This in turn could result in greater motivation for self-enhancement in easy tasks than in difficult tasks. Therefore, although the cognitive comparison to the average or typical student might be “correct,” the selection of the comparison standard might be motivated by self-enhancement. The reasons for the results of the present study are another topic for future studies.

In short, although the BAE is the most widely cited piece of evidence for self-enhancement bias in comparative ability judgments, the BAE may not be a valid measure of self-enhancement because people do not always interpret “average ability” as median ability. Despite the seemingly pervasive evidence for the traditionally defined BAE, the extent to which people inflate their self-evaluation of abilities might have been overstated in the literature. To document the presence of self-enhancement bias in comparative ability judgments, future research needs to consider the meanings people assign to “average ability” in specific ability domains.

## Author Contributions

Y-HK: generating the research ideas and hypotheses, collecting the data, preparing the manuscript. Y-HK, HK, C-YC: analyzing the data, making alternations to the last version of the manuscript.

## Conflict of Interest Statement

The authors declare that the research was conducted in the absence of any commercial or financial relationships that could be construed as a potential conflict of interest.
